# Improving healthcare empowerment through breast cancer patient navigation: a mixed methods evaluation in a safety-net setting

**DOI:** 10.1186/1472-6963-14-407

**Published:** 2014-09-19

**Authors:** Guzyal Gabitova, Nancy J Burke

**Affiliations:** Global Health Sciences, University of California San Francisco, San Francisco, CA USA; Department of Anthropology, History, and Social Medicine and Helen Diller Family Comprehensive Cancer Center, University of California San Francisco, San Francisco, CA USA

**Keywords:** Patient navigation, Breast cancer, Disparities, Healthcare empowerment

## Abstract

**Background:**

Breast cancer mortality rates in the U.S. remain relatively high, particularly among ethnic minorities and low-income populations. Unequal access to quality care, lower follow up rates, and poor treatment adherence contribute to rising disparities among these groups. Healthcare empowerment (HCE) is theorized to improve patient outcomes through collaboration with providers and improving understanding of and compliance with treatment. Patient navigation is a health care organizational intervention that essentially improves healthcare empowerment by providing informational, emotional, and psychosocial support. Patient navigators address barriers to care through multilingual coordination of treatment and incorporation of access to community services, support, and education into the continuum of cancer care.

**Methods:**

Utilizing survey and qualitative methods, we evaluated the patient navigation program in a Northern California safety-net hospital Breast Clinic by assessing its impact on patients’ experiences with cancer care and providers’ perspectives on the program. We conducted qualitative interviews with 16 patients and 4 service providers, conducted approximately 66 hours of clinic observations, and received feedback through the self-administered survey from 66 patients.

**Results:**

The role of the patient navigator at the Breast Clinic included providing administrative assistance, psychosocial support, improved knowledge, better understanding of treatment process, and ensuring better communication between patients and providers. As such, patient navigators facilitated improved collaboration between patients and providers and understanding of interdisciplinary care processes. The survey results suggested that the majority of patients across all ethnic backgrounds and age groups were highly satisfied with the program and had a positive perception of their navigator. Interviews with patients and providers highlighted the roles of a navigator in ensuring continuity of care, improving treatment completion rates, and reducing providers’ workload and waiting time. Uncertainty about the navigator’s role among the patients was a weakness of the program.

**Conclusions:**

Patient navigation in the Breast Clinic had a positive impact on patients’ experiences with care and healthcare empowerment. Clarifying uncertainties about the navigators’ role would aid successful outcomes.

## Background

Breast cancer mortality rates among women living in the United States remain the second highest among all types of cancer [1]. Breast cancer is a curable disease if detected early and treated in a timely manner. However, significant disparities in breast cancer mortality in the U.S. still exist as many studies have shown that both breast cancer incidence and mortality rates vary by ethnicity and socioeconomic status [2, 3]. Previous studies proposed several factors that contribute to ethnic disparities in receiving quality cancer care, particularly among breast cancer patients. They include a higher likelihood of being diagnosed at a later stage, lower probability of having positive tumor receptors for estrogen and progesterone, [2] lower likelihood of receiving chemo- and radiation therapy, [4] unequal access to timely adequate cancer care, [5] inadequate follow-up of abnormal mammogram, [6] lower treatment adherence rates, [7, 8] higher obesity, [9] and lower literacy rates [10]. Negative treatment outcomes can also be attributable to poverty and lack of insurance [8, 11].

To address disparities in access to, quality of, and use of care among ethnic minorities as outlined in the 2002 Institute of Medicine Report, [12] the US Congress passed the Patient Navigator Outreach and Chronic Disease Prevention Act in 2005. According to this act, patient navigation was envisioned as an effective intervention to improve health outcomes by reducing delays to quality care through the use of a person guide throughout the healthcare system [13]. Patient navigation represents a patient-centered approach that reduces patient, provider, and health system barriers for the majority of at-risk populations by incorporating coordination of treatment and services, emotional support, and education into the continuum of cancer care [14]. Since the mid1990s, many U.S. hospitals have implemented patient navigation programs to improve follow-up and treatment rates for cancer among medically underserved populations [15]. Although studies have shown that navigation improves patients’ experiences and satisfaction with care, [16–20] very few have evaluated the clinical efficacy of such programs, particularly their long-term impact on the adherence to treatment [8] and survival [21]. None, to our knowledge, have evaluated the link between patient navigation and healthcare empowerment.

### Healthcare empowerment

Patient empowerment, as theorized in health services and clinical research, rests upon the ability of the *individual patient* to acquire the negotiation and navigation skills, be they communication- (e.g. question-asking skills) or computer-based, [22–25] to take responsibility for their own healthcare. A number of researchers have hypothesized that giving patients access to their own electronic medical records could be what is needed to address low levels of patient empowerment. The concept of Healthcare Empowerment (HCE) begins to broaden the frame from such discrete, individual-level interventions to note the healthcare system and social factors relevant to empowerment. Rather than residing within the individual (e.g. “patient” empowerment), HCE is achievable at the intersections of individuals, communities, and healthcare organizations. HCE, as such, is defined as the process and state of participation in health care that is characterized as (1) engaged, (2) informed, (3) collaborative, (4) committed, and (5) tolerant of uncertainty [26]. However, there are important cultural variations in the role that one is encouraged or expected to play in one’s own health care. HCE, following this definition, is influenced by the context in which individuals live, including socioeconomic status, cultural background, gender norms, and generational factors; and the background, training, culture, expectations and pressures of providers [26]. HCE is theorized to improve outcomes due to patient collaboration with providers and commitment to completion of treatment [26]. Patient navigation, as a healthcare organizational intervention, addresses key aspects of HCE, creating a ‘relational bridge’ between patients of diverse ethnic, cultural, social, and economic backgrounds and the professional culture of interdisciplinary breast cancer care. In the following, we describe the ways in which patient navigators in an Urban Hospital breast clinic engage in practices that have implications for the healthcare empowerment of their patients.

### Breast cancer patient navigation in a safety-net setting

The Breast Cancer Patient Navigation Program at Urban Hospital^a^ started in mid-1997 in response to increasing needs of underserved women with breast cancer in the local urban area. Navigators at the Breast Clinic are lay health workers with no clinical background who do not possess specific qualifications except for some prior experience in a hospital setting, strong communication skills, and ability to work in a complex, multi-cultural setting with vulnerable patients. Bi-or multilingualism is essential for a subset of navigators since a high proportion of patients are monolingual in Spanish, Cantonese, Mandarin, Vietnamese, Russian, and more than 20 other languages. This linguistic and cultural expertise distinguishes this safety-net navigation model as programs elsewhere employ nurses, [16, 27] survivors, [20] or social workers [17] to navigate patients. Another distinction of the program is the fact that virtually every breast cancer patient, regardless of ethnicity, language, or income, is assigned a navigator in the first day they enter the clinic, at times prior to their diagnostic resolution. At the time the study was conducted, the safety-net breast clinic employed five patient navigators. Navigators offer a full range of services to address each patient’s medical, emotional, psychosocial educational and support needs. They provide their services in English, Spanish/Portuguese and Cantonese/Mandarin.

## Methods

We conducted a mixed method study designed to assess the effectiveness of the patient navigation program in Urban Hospital’s Breast Clinic. The goal of our study was to identify the strengths and weaknesses of the patient navigation program by evaluating its impact on patients’ HCE through improved access to and engagement with quality standard care, patient experience of breast cancer care and communication with providers, relationship with their patient navigator and the broader care team, and provider perception of and reliance upon the patient navigation program. Our study design included a patient self-administered multi-lingual questionnaire (e.g. English, Spanish, Chinese [short form], and Russian), in-depth qualitative interviews with patients and providers, and participant observation at the Breast Clinic during and after which the first author recorded detailed field notes about clinic flow, patient/navigator interactions, patient/provider/navigator interactions, and provider/navigator interactions once a week for 11 weeks, thereby, observing 11 clinic days, for a total of 66 hours.

Our study population included 83 patients who were assigned a navigator during their treatment at the breast clinic (67 completed a mail-in self-administered survey – 20 Chinese [short form], 9 Spanish, 5 Russian, 32 English; and 16 patients participated in in-depth qualitative interviews – 3 Cantonese, 3 Spanish, 3 Russian, 7 English) and 4 providers who interacted with patients and their navigators.

### Sampling and recruitment

Together with the coordinator of the Breast Cancer Patient Navigation Program we identified potential self-administered survey participants based on a database that included all breast cancer patients who had a navigator in the year 2010 and 2011 and had been enrolled in the navigation program for at least six months by the time of the interview.

Patients were considered ineligible if they had severe mental health or substance abuse issues. We used convenience sampling to select 161 patients who met eligibility criteria and mailed the survey to all patients in April 2012.

We used purposive sampling to identify eligible patients for qualitative interviews. Because we wanted to interview patients of the four major language groups represented in the clinic, we selected Spanish-, Cantonese-, Russian-, and English-speakers. All but Russian-speaking patients were assigned navigators who spoke their first language. This difference allowed us to explore patient perceptions of the importance of language and ethnic concordance in the interviews.

After we identified eligible patients, the first author approached English- and Russian-speaking patients over the phone or in person during their appointment in the clinic and asked whether they would be interested in participating in a 45–60 minute qualitative interview about their experiences with the navigation program. Bilingual, bicultural research team members recruited Spanish- and Cantonese-speaking patients. If the patient agreed, we scheduled an appointment at their convenience. If they refused, we moved on to the next eligible candidate. Five patients refused to participate due to time conflicts, absence or illness. Patients were considered eligible if they were 18 years or older, able to provide informed consent, had a confirmed diagnosis of carcinoma in-situ or invasive carcinoma regardless of stage and tumor type, and were able to understand written English, Spanish, Russian or Chinese [short form]. Patient interviews focused on communication with a navigator and other providers, personal relationship with the navigator, desired services, examples of the most helpful services provided by the navigator etc. We adapted the interview guide used in a similar study elsewhere [20]. All patient interviews took place at the hospital cafeteria, patients’ homes or coffee shops.

Our survey aimed to evaluate the impact of the navigation program on patients’ healthcare empowerment by assessing access to quality cancer care, experience of breast cancer treatment, relationship with their navigator, services received, and satisfaction with the navigation program in general. After conducting an extensive literature review, we identified several scales appropriate for our study, and adapted measures for low literacy [16, 18]. We pilot tested the survey with 3 patients and 2 patient navigators, and then translated it into Spanish, Chinese [short form] and Russian. We mailed the survey along with a letter introducing the study, an implied consent form, and a prepaid return envelope to 161 patients in April 2012. All patient survey responses were entered into REDCap version 4.8.4(21), a secure application for managing surveys and databases. We also purposively identified four healthcare providers who worked closely with the Navigation Program: a medical oncologist, two nurse practitioners and a physician’s assistant, and invited them to participate in a 45–60 min interview by phone or email. Provider interviews focused on overall perception and perspectives of the patient navigation program, view of the navigator’s role, and impact on patients’ treatment process, engagement and knowledge.

All interviews were digitally recorded and transcribed verbatim by certified translators and one of the investigators. We used qualitative data analysis software, Atlas.ti Version 6.2.23, to facilitate coding and analysis of interview and field observation data.

Each patient participant received a $25 Target gift card. Service providers did not receive an incentive. The university’s Committee on Human Research approved all study procedures on March 14, 2012 (IRB# 12–08494); all individuals signed informed consent prior to their inclusion in the study.

## Results

### Survey

We received a total of 66 completed surveys, achieving a 41% response rate. Demographic characteristics of the respondents are shown in Table 1. The mean age of the study sample was 55, nearly half were Asian, and a majority indicated English as their primary language, followed by Cantonese or Mandarin. Most were married or had a partner and were employed at the time of diagnosis.

**Table 1:** **Baseline demographics of 66 survey respondents**

Characteristic	N	%
**Age**		
30-39	6	9
40-49	13	20
50-59	23	35
60-69	21	32
70<	3	5
**Ethnicity**		
Asian	32	48
White	11	17
Hispanic or Latino	9	14
Black/African American	8	12
American Indian/Alaska Native	1	2
Other	4	6
**Primary language**		
English	26	39
Cantonese or Mandarin	20	30
Spanish	7	11
Russian	5	8
Other	7	11
No response	1	2
**Marital status**		
Married or living with a partner	25	38
Never married	18	27
Divorced, widowed or separated	21	32
No response	2	3
**Education**		
No formal education	1	2
High school or less	34	52
College/Vocational or technical school	24	36
Graduate or professional degree	4	6
No response	3	5
**Employment**		
Employed	30	45
Unemployed	19	29
Student	2	3
Retired	9	14
Other	6	9
**Annual household income**		
$0 to $9,999	20	30
$10,000 to $14,999	12	18
$15,000 to $19,999	11	17
$20,000 to $34,999	12	18
$35,000 to $49,999	4	6
$50,000 to $74,999	1	2
No response	6	9
**Health insurance**		
Yes	51	77
No	13	20
No response/Don’t know	2	3
**Number of children**		
No	20	30
1 – 2	26	39
3 – 4	14	21
5 or more	1	2
No response	5	8

### Services received from the navigator and desired services

Patients were asked to indicate the services that they received from their navigator and services they needed, but did not receive. The services that received the highest score were financial assistance (62%), followed by transportation (53%), help with medical insurance (48%), appearance (47%) and referrals to alternative therapies (45%). Additional services included emotional support, assistance with paperwork and help with scheduling medical appointments. None of the respondents reported receiving assistance with employment or childcare. Some women felt they needed assistance with transportation (17%) and financial support (14%) that was not received (Table 2).

**Table 2:** **Services received from the navigator and desired services**

	Received		Desired	
	N	%	N	%
Finding a primary care doctor	16	(24)	5	(8)
Medication	22	(33)	3	(5)
Appearance	31	(47)	2	(3)
Clothing	17	(26)	5	(8)
Referrals to alternative therapies	30	(45)	6	(9)
Employment	0	0	6	(9)
Childcare	0	0	4	(6)
Financial assistance	41	(62)	9	(14)
Housing	6	(9)	6	(9)
Information about sexual intimacy	1	(2)	3	(5)
Legal services	5	(8)	2	(3)
Medical insurance	32	(48)	4	(6)
Transportation	35	(53)	11	(17)
Translation assistance	27	(41)	5	(8)
None	2	(3)	17	(26)
Other	9	(14)	2	(3)
No response	2	(3)	21	(32)

### Patient satisfaction

We included 18 statements in the questionnaire that were intended to measure the patients’ overall satisfaction with the navigation program. Patients were asked to rate each statement with a four-item Likert-type scale that included *Strongly agree*, *Somewhat agree*, *Somewhat disagree and Strongly disagree*. To analyze the responses, we dichotomized the scale as ‘*Agree*’ and ‘*Disagree*’. Overall, patients’ responses demonstrated high levels of satisfaction with the navigation program. The vast majority of patients had a positive perception of their navigator: more than 90% of the patients agreed that their navigator was friendly and respectful. Slightly fewer patients felt that their navigator was sensitive (74%). Availability of the navigator and timely callback also received a high score (94% and 91% respectively).

A key aspect of healthcare empowerment is access to appropriately framed and delivered information. In general, navigators met patients’ informational needs. About 80% agreed that their navigator kept them informed, explained their diagnosis and information provided by other health professionals. Over half of patients received question-asking support (67%) and over three fourths received help with finding health-related information (77%). Most patients were satisfied with emotional support provided by navigators (~80%); only 14% wanted to switch their navigator. A high proportion of patients (83%) rated the program as ‘*Excellent*’, ‘*Very good*’, *or* ‘*Good*’. Fifteen percent rated it as ‘*Poor*’ *or* ‘*Fair*’. Those patients who gave the program a lower score either did not have a good experience with their navigator or did not specify the reason why they thought the program was not helpful.

### Qualitative interviews

Sixteen women participated in in-depth, in-person qualitative interviews. The mean age of participants was 54, and our sample was one-third Asian; one-third non-Hispanic White, the rest being Latina and African American women. The majority spoke English as their primary language; the rest were equally divided between Spanish, Cantonese and Russian (Table 3).

**Table 3:** **Baseline demographics of 16 qualitative interview participants** (**patients**)

Characteristic	N	%
**Age**		
40-49	5	31%
50-59	8	50%
60-69	2	13%
70 >	1	6%
**Ethnicity**		
White, non-Hispanic	5	31%
Asian (Chinese)	4	25%
Asian (Filipino)	2	13%
Hispanic	3	19%
African American	2	13%
**Primary language**		
English	6	38%
Cantonese	3	19%
Spanish	3	19%
Russian	3	19%
Tagalog	1	6%
**Marital status**		
Married/Living with a partner	7	44%
Never married	5	31%
Divorced	3	19%
Widowed	1	6%
**Current occupation**		
Employed	6	38%
Unemployed	7	44%
Student	2	13%
Retired	1	6%
**Number of children**		
None	6	38%
1 or 2	9	56%
3 and more	1	6%
**Length of navigation**		
Less than 1 year	4	25%
1-2 years	5	31%
3-5 year	6	38%
More than 5 years	1	6%
**Tumor type**		
Invasive ductal carcinoma	8	50%
Ductal carcinoma in-situ	3	19%
Invasive lobular carcinoma	1	6%
Unknown	4	25%

A wide range of codes were utilized in the analysis of our qualitative data. In the following we report themes that emerged relating patient navigation to healthcare empowerment.

### Continuity of care

Breast Clinic at Urban Hospital is an interdisciplinary medical setting where patients often see a different provider at each appointment. Therefore, one of the fundamental roles of patient navigators is to ensure continuity of care for patients in this overwhelming environment, as they are the one provider that stays constant. As noted by one of the patient interviewees,

“*How would the patient know what to do*? *How would they know the ropes*? *Whoever thought of the navigation is a genius. My navigator has been like an anchor to me*, *because you are being seen by a lot of doctors. She would talk to them*, *and help me to get things done in the most convenient way*”. (*Patient 12*, *Filipina*)

As this quote indicates, patients perceived their navigator as a connecting bridge between themselves and the multidisciplinary healthcare team of the breast clinic, providing them with stability and comfort in this potentially complicated environment. Such sense of stability enabled patients to take a collaborative stance and actively engage in their treatment process, both of which are proposed elements of HCE.

Navigators coordinate treatment appointments outside of Urban Hospital as well, including accompaniment to radiation appointments at the affiliated academic hospital and connections with community based organizations providing financial, housing, and psychosocial support. As a Nurse Practitioner noted,

“*There is bad communication between radiation oncology at* [*affiliated academic hospital*] *and Urban Hospital. When patients go to the appointments at radiation oncology we don*’*t even know what happened there*; *the navigators usually report what happened to us. Of course*, *we can*’*t take their word for it*, *it has to be documented*, *but they actually get a report and bring it back* [*to the breast clinic*]. (*Provider 1*, *nurse practitioner*)

### Treatment engagement

Commitment to one’s health and treatment plan is a key concept of HCE [26]. All providers in our study reported that the navigation program improved treatment adherence and completion rates primarily due to the practice of calling patients to remind them of scheduled appointments (e.g. chemotherapy, radiation or follow up at the breast clinic). Navigators become very involved with their patients on a personal level, which providers felt also influenced compliance. It was particularly evident with patients at higher risk for non-compliance,

“*There are differences between the patients who have a navigator and those who do not if we factor in mental health issues and substance abuse. I had a young patient who*’*s got an addiction problem and mental issues. She was lost to follow up but continued to receive calls from her navigator. Maintaining that relationship brought the patient back to treatment. And that*’*s the difference. Adherence is multifactorial*; *it*’*s difficult to address it. But what is a better measurement is that those people who are not adherent and then re*-*enter the system*, *usually do it through the navigator*”. (*Provider 2*, *nurse* -*practitioner*)

On multiple occasions we observed the ways through which navigators ensured that every one of their patients was present at their appointment. If the patient was missing, the navigator followed up by phone right away. Navigators’ role in reducing loss to follow-up and improved adherence was noted by the providers interviewed,

“*I feel good knowing that the patient is more likely to stay on track*, *to be compliant*; *whether they are compliant on their own or because they have a navigator. Whatever the case might be*, *the rate of canceled cases associated with breast surgery is significantly less than other cases. So there is something to say for that*”. (*Provider 3*, *nurse practitioner*)

### Reducing providers workload and waiting time

Ineffective communication and utilization of providers’ time in the clinic can negatively affect the development of HCE. In fact, long waiting times were a common source of frustration for many patients. Navigators help to decrease waiting time at the clinic by taking on some of the non-medical roles of providers. Most common things mentioned by providers and patients included scheduling appointments, arranging scans and other medical procedures, and dealing with associated paperwork and health insurance. Navigators are the first point of contact for the patients; patients reported calling the navigator to ask questions or if they needed to cancel or reschedule an appointment, which also takes a great deal of pressure off the providers’ shoulders. Every interviewed provider appreciated this aspect of the navigators’ work,

“*I don*’*t have time to answer patients*’ *calls unless I*’*m the one who initiates the call. For me navigators are insulations from those calls. So it*’*s invaluable*”. (*Provider 4*, *medical oncologist*)

This relationship also created a sense of connection and access to the clinic: a patient could always call the navigator who would either answer the phone or call back later. Knowing that there was someone to answer their questions and to listen was important to patients and provided relief to providers.

### Improved patient-provider communication

Assertive communication and constructive listening skills are the personal resources that are hypothesized to be associated with HCE. Foreign-born patients with a limited command of English constitute a large proportion of the patient population at Urban Hospital, and their ability to communicate effectively with their providers and thus actively engage in their treatment process might be diminished. Hence, many interviewees felt that navigators influenced their ability to ask questions in a positive way. When the patient was monolingual, the navigator would talk to them before seeing the provider, write all their questions down and ask the provider on behalf of the patient,

“*Sometimes when I don*’*t know how to ask a doctor*, *I would ask* [*my navigator*] *how I should ask. She would tell me* –*say it just like this. So then*, *I would ask that way when I see my doctor. Sometimes*, *I don*'*t know how to phrase my questions in a way the doctor will understand*, *so I check with my navigator first. For instance*, *I*'*d say*, "*If I want this*, *how should I ask the doctor*?" *Then*, *she gives recommendations on how to phrase my questions*”. (*Patient 16*, *Chinese*)

When language was not a barrier, the navigator listened to the patient’s concerns and offered examples of what kind of questions they might want to ask the doctor. Providers reported the importance of the role of navigators in facilitating question asking, noting that for some patients it might be challenging to ask medical questions due to cultural norms or prior negative experiences,

“*A lot of our patients come from cultures when you don*’*t dare to question a* ‘*mighty doctor*’. *And stuff doesn*’*t get addressed. And the navigators help with that*, ‘*We were talking about something a little while ago*, *why don*’*t we talk about that with the doctor*?’” (*Provider 4*, *medical oncologist*)

Many patients, regardless of their first language, reported difficulty understanding medical terminology. In this context, the navigator’s role in clarifying medical language and speaking in understandable, clear language was reported by a number of patients and providers as one of the most valuable aspects of the navigator program.

“*Even though English is not my navigator*’*s native language she speaks terminology that I can understand*, *while oncologists speak percentages and other technical stuff*, *which I can*’*t even hear sometimes. Navigators are much simpler to understand. She is not wearing a white coat so you don*’*t feel medical with her*”. (*Patient 1*, *White*)

Navigators often spend some time with patients before a provider comes into the room or after the appointment. They clarify questions about patients’ diagnosis and treatment, required diagnostic procedures or interpret the results of such tests. They usually dedicate more time to first-time patients or someone who they feel needs more in-depth explanation or support. Providers generally expressed trust in navigators’ knowledge and ability to provide accurate health-related information.

“*I don*’*t think the navigators are making things up*, *sometimes they are explaining in more patient*-*friendly terms*, *for instance*, [*the provider might say*], ‘*You will have sentinel node biopsy*.’ *But the patient might not understand. And the navigator will explain*, ‘*That means that we will inject a dye etc*.’ *There are very few providers who will go to that level of detailed explanation. You get fellows and residents come in and talk to patients*, *and they are very new in their practice*, *they don*’*t know very much about it. And people who have been doing it for a long time can be very jaded and tired*; *they have a different approach*: *we*’*ll just deal with that later. And that later part is a navigator. They are interpreting medical language*”. (*Provider 2*, *nurse practitioner*).

As these quotes suggest, navigators facilitate HCE through improving their patients’ question-asking and communication abilities in the medical setting both for monolingual and English speakers. We also observed that navigators provided essential information about the patient to the provider, information which would not be possible to obtain by looking at the patient’s chart alone. Such information then informed decision-making regarding treatment and scheduling. Providers interviewed suggested that this broader view of the patient improved their ability to match treatment plans with patient needs, and therefore potentially led to more effective health outcomes.

### Emotional support

Emotional distress, uncertainty, and fear can undermine patient HCE. Many patients in our study reported emotional distress due to their disease being greater than the physical pain associated with surgery and treatment. Patients mentioned emotional support provided by their navigator as one of the most important aspects of the navigation program,

“*Anything I need I can call my navigator. She*’*s good. She makes me feel good*, *she gives me strength*”. (*Patient 3*, *African American*)

Having a navigator was also a stress-relieving factor for many participants because navigators took care of administrative responsibilities, such as dealing with medical insurance, housing issues and medical bills. Each provider we interviewed noted that having a navigator in the room provided comfort for the patients and made them feel emotionally relaxed.

“*I usually see my navigator at the breast clinic and every time I see her if I need information and I*’*ll chat with her and she*’*ll answer all my questions*, *she*’*ll help me to deal with the appointments and will make sure it*’*s all set. She gives me peace of mind and that*’*s already taking care of my stress*”. (*Patient 4*, *Asian*, *English speaking*)

Among the patients who participated in interviews only one woman reported being unhappy with the lack of emotional support from her navigator.

### Health education/information

Navigators at Urban Hospital Breast Clinic are not specifically asked to provide health education beyond explaining and interpreting information delivered by providers. However, in response to training received through an ongoing Navigator Education Series offered by Urban Hospital to the Breast Clinic Navigators and their community partners, navigators provide patients with information about things like diet and physical activity.

However, several patients also reported lack of explanation from their navigator. One patient felt that her navigator did not clearly explain the need for having a chemotherapy port (or a ‘PICC line^b^’). The patient subsequently refused the procedure, which she felt resulted in a mild injury to her arm from too many injections,

“*If* [*the navigator*] *had explained more clearly*, *or told me that I can either have the procedure on my arm versus near the heart*, *I would have made a better decision. I*’*m not an animal*, *I have my own thoughts. I think the role of* [*the navigator*] *is to use her knowledge to educate the patient so the patient can make the appropriate decision*”. (*Patient 15*, *Chinese*)

Although in general providers expressed confidence in navigators’ ability to communicate health-related information to the patients, one of them noted some limitations in their knowledge when they worked with complicated cases,

[*Navigators*] *are not so good if you vary the treatment often. If you move to the other arena they can get confused. That*’*s when they go on autopilot a little too much. They have to really understand why we are doing the test or prescribing specific medicine*. (*Provider2*)

These examples demonstrate the importance of ongoing education and communication to navigators’ ability to enhance patient HCE.

### Lack of clarity about the navigator’s role

Uncertainty about the navigator’s role was discussed in all patient interviews. When patients come to the breast clinic for the first time they are not familiar with its structure. Moreover, they are often too overwhelmed to fully comprehend the situation. In the midst of this new experience, many patients reported not understanding the role of the navigator, who she was, and why she was in the room.

One English-speaking Asian patient thought her navigator was assigned to her to translate because she was Chinese. Another patient said that after seven months, she still was not sure about the role of the navigator. In fact, because she did not know the navigator and her function, she felt that her privacy was somehow violated,

“*I wish she took her time to tell me*, ‘*I*’*m your navigator*, *these are the things that I need to do for you*’, *so I would understand. She didn*’*t introduce herself or her role. She just said* - *I*’*m your navigator. If you don*’*t tell me what your role is as a navigator*… *I guess I felt that my privacy was open to the navigator. Because I don*’*t know what the role of the navigator was*”. (*Patient 7*, *Filipina*)

In general, patients reported feeling comfortable with the navigator being in the room while they talked with their providers and helping them during their treatment, but the majority of patients did not understand the scope of services provided by the navigator. According to providers, confusion about the navigators’ role is common in clinic, particularly among new patients and even new staff. When patients did not understand the purpose and extent of the navigation program, they were not able to utilize its full potential.

## Discussion

There are many gaps in our understanding of how patient navigation works. To eliminate gaps in research, the National Cancer Institute launched a Patient Navigation Research Program (PNRP), whose goal was to evaluate patient navigation across nine sites in the United States. These studies utilized an experimental design in order to determine the efficacy and cost-effectiveness of patient navigation [14]. PNRP developed a definition of navigation and instruments to effectively evaluate the findings of these studies [14, 18].

Preliminary results of the nation-wide research project suggest that navigation significantly decreased time to diagnostic resolution for patients with cervical and breast screening abnormalities [28–31]. However, in some studies this effect became apparent only after 30 days [31] or six months from the detection of the abnormality [29]. Another PNRP study revealed that navigation services helped improve satisfaction with care among certain disadvantaged groups of the population [32]. The goal of our study was to assess and define the model of the patient navigation program at Urban Hospital Breast Clinic by evaluating its impact on patients’ HCE, experiences with cancer care, and providers’ perspectives on the program. Although there were variations in roles and responsibilities of the individual navigators within the program, most of their tasks were similar and included linking patients to community resources, assisting with medical insurance, keeping track of electronic medical records and appointments, providing emotional support, interpreting in the patient’s primary language, and facilitating patient-provider communication during appointments. Navigators met patients’ informational needs by providing health education and clarifying medical language used by providers. They also fulfilled a number of logistic duties, such as scheduling follow up appointments, arranging transportation, and providing access to financial resources. This ‘scope of work’ is consistent with what navigators elsewhere were engaged in [16, 20, 33]. A 2014 literature review notes that the primary role of navigators in some programs also included addressing barriers to cancer clinical trial enrollment for minority patients [34]. This was not the case at Urban Hospital. Navigators were regularly present during clinical trial recruitment discussions, however, and were often asked to interpret, allowing monolingual minority patients to explore treatment options that would not be available to them otherwise, and giving medical researchers more opportunities to recruit participants from more diverse backgrounds. Results of our quantitative survey suggest that a large proportion of patients were highly satisfied with the navigation program and the services that they received from their navigator, with no urgent demand for any specific services.

There is currently no standardized model of navigation in breast cancer care; every navigation model is unique and has its distinctions. A qualitative evaluation of a public hospital breast cancer navigation program that employed breast cancer survivors as navigators found that the survivorship component was one of the key features of the program for patients as their navigator served as evidence that surviving breast cancer was possible [20]. Another study suggested that “oncology nurses are particularly well positioned to advocate for the navigator role,” [27] probably due to their clinical background and position at the hospital. However, the fact that all navigators in Urban Hospital’s program were lay persons was seen as an advantage by most patients since it facilitated their communication of medical information in clear language and minimized perceived class or professional differences (e.g. “they don’t wear white coats”). Hiring lay persons as navigators also made it easier to bring together a multilingual team, which is not a common feature of navigation programs elsewhere [35].

Although we have observed and documented benefits of the program across all age groups and ethnicities, the program was particularly valuable for monolingual patients who had a navigator who spoke their first language (Chinese/Mandarin and Spanish/Portuguese speaking patients). Navigators acted as informal medical interpreters during appointments (usually if the patient required a professional interpreter – the clinic would provide telephone interpretation, which, according to patients, is not a convenient way of communicating and often adds more stress). Additionally, taking into account that all monolingual patients were foreign-born to who the US medical system was unfamiliar, having a navigator allowed them to smoothly enter the system and stay on track with treatment.

Both providers and patients in our study noted the key informational and communication role played by navigators. Ability to address informational needs is a key aspect of HCE [26]. These communication needs were met through the translation of medical language into understandable concepts, listening to patients, identifying their questions, and assisting in question-asking of providers. Navigators spent time with patients before and after their appointments, and often accompanied patients to unfamiliar institutional contexts to complete radiation or other diagnostic scans. This ‘extra’ time and consistency of attention enabled navigators to establish relationships with many patients; a particularly important and empowering aspect of such a program in a rushed, sometimes chaotic clinic setting where patients rarely see the same provider twice. This trust is augmented by the navigator’s attention to the patient’s broader social, financial, and emotional needs. In addition to trust, this extra time and the information gathered and held by the navigators supports improved efficiency of appointment time utilization.

Not all patients and providers fully utilized the benefits of the navigation program, however, due to uncertainty about the scope of the navigator’s role and inconsistency in clear communication of this role. Although every provider interviewed understood the purpose of the navigation program, some patients did not have a clear idea of the roles and responsibilities of their navigator and program goals. In some instances, such lack of understanding resulted in patients’ needs not being met. Clarification of the navigator’s role in the initial stages of the treatment process would significantly simplify and improve patients’ experience with the program and help avoid miscommunication in the future. For novice providers in the clinic, introduction to the program and recognition of the navigator as another member of the team should become a common practice.

Several interviewees suggested that creating a brochure or a leaflet that describes the role of the navigator and the program in general would help to utilize the services more efficiently. Indeed, the informational package is something that would be helpful for women who get introduced to the clinic for the first time since first appointments are usually too overwhelming and emotional for most patients to remember anything that was verbally delivered to them, including the description of the navigation program.

### Limitations

Using convenience sampling to select survey participants was one of the limitations of our study, which restricts the generalizability of results to other hospitals, types of cancer or male patients. Patients with known mental illness or other cognitive impairments were omitted from the survey sample. While these patients receive navigation, their perspectives were not included in the data. Lack of randomization limited assessment of the impact of the program on patients’ clinical outcomes.

In addition, while we were able to explore the insights of other major ethnic groups through the qualitative in-person interviews, the ethnic makeup of our survey respondents was predominantly represented by Asian females (48%). This reflects the city’s demographics as Asians remain its largest single ethnic minority group at 30% [36].

### Recommendations

Considering the importance of medical interpretation during the appointments and to avoid medical errors, [37] it might be beneficial to combine credentialing as a medical interpreter with patient navigator training in safety-net settings such as Urban Hospital. In addition, since underserved patients served in safety-net settings more often are afflicted with psychological and substance abuse problems, providing patient navigators with additional training in psychosocial counseling and direct access to a staff psychologist mentor may improve the effectiveness of such a program.

## Conclusions

Results of our analysis suggest that patient navigation at Urban Hospital Breast Clinic had a positive impact on breast cancer patients’ HCE and experiences with care. The majority of patients perceived that navigation improved their communication with providers and provided administrative assistance, better knowledge, encouragement and psychosocial support. Although not unique in its research design, this study was the first evaluation of the patient navigation program at Urban Hospital since its foundation in 1997. The findings were presented to the staff of the Breast Clinic in August 2012 and will serve as a basis for enhancement and possible expansion of the program. Indeed, in response to the August 2012 presentation, the program developed an information sheet, translated into three languages, describing the scope of the patient navigator’s role which is provided to new clinic patients.

Continuous evaluation of the program is essential to ensure quality improvement. Further research in this particular setting to assess adherence to treatment and cost-effectiveness of the program is needed. Taking the navigators’ perspectives on the program might expose more areas for potential improvement.

## Endnotes

^a^The name of the hospital under study has been changed to protect anonymity of research participants.

^b^Peripherally inserted central catheter (PICC line) is a tube that is inserted into a peripheral vein, normally in the upper arm, to obtain intravenous access for a prolonged period of time.

## Abbreviations

HCE: Healthcare Empowerment
PNRP: Patient Navigation research Project.
